# Correlation between the triglyceride-glucose index and arterial stiffness in Japanese individuals with normoglycaemia: a cross-sectional study

**DOI:** 10.1186/s12902-024-01551-2

**Published:** 2024-03-05

**Authors:** Yuying Cai, Wenyue Sha, Hailian Deng, Tuming Zhang, Linlin Yang, Yueying Wu, Jinhua Luo, Guangyan Liu, Yu Yang, Dehui Feng

**Affiliations:** Affiliated Hospital of Guangdong Medical University, No. 57 Renmin Street, 524000 Zhanjiang, China

**Keywords:** Triglyceride-glucose index, Arterial stiffness, Normoglycaemia

## Abstract

**Background:**

The association between the triglyceride-glucose (TyG) index and arterial stiffness in individuals with normoglycaemia remains unclear. We aimed to evaluate the relationship between the TyG index and arterial stiffness in Japanese individuals with normoglycaemia, providing additional evidence for predicting early arterial stiffness.

**Methods:**

This study included 15,453 adults who participated in the NAGALA Physical Examination Project of the Murakami Memorial Hospital in Gifu, Japan, from 2004 to 2015. Data on clinical demographic characteristics and serum biomarker levels were collected. The TyG index was calculated from the logarithmic transformation of fasting triglycerides multiplied by fasting glucose, and arterial stiffness was measured using the estimated pulse wave velocity calculated based on age and mean blood pressure. The association between the TyG index and arterial stiffness was analysed using a logistic regression model.

**Results:**

The prevalence of arterial stiffness was 3.2% (500/15,453). After adjusting for all covariates, the TyG index was positively associated with arterial stiffness as a continuous variable (adjusted odds ratio (OR) = 1.86; 95% Confidence Interval = 1.45–2.39; *P*<0.001). Using the quartile as the cutoff point, a regression analysis was performed for arterial stiffness when the TyG index was converted into a categorical variable. After adjusting for all covariates, the OR showed an upward trend; the trend test was *P*<0.001. Subgroup analysis revealed a positive association between the TyG index and arterial stiffness in Japanese individuals with normoglycaemia and different characteristics.

**Conclusion:**

The TyG index in Japanese individuals with normoglycaemia is significantly correlated with arterial stiffness, and the TyG index may be a predictor of early arterial stiffness.

## Background

Cardiovascular diseases (CVDs) are a major cause of disability and mortality worldwide [1]. Arterial stiffness is one of the earliest functional impairments of the vascular aging process that directly affects the cardiovascular system through loss of vascular reactivity and an increase in vascular thickness and stiffness [2]. Arterial stiffness is an independent predictor of adverse cardiovascular events [3]. However, the biological mechanisms underlying arterial stiffness remain unclear. Early identification of predictors in patients allows appropriate preventive measures, thereby reducing the incidence of CVDs and the global healthcare economic burden.

There are various methods for assessing arterial stiffness, such as the pulse wave velocity (PWV), dynamic arterial stiffness index (AASI), arterial pulse waveform (enhanced index), and magnetic resonance imaging. However, their measurement has limitations, such as high technical requirements, specialised equipment, and high cost, and it is not widely used in clinical practice [4]. Recent studies have shown that estimated pulse wave velocity (ePWV) calculated based on age and mean blood pressure (MBP) is a new indicator that can reliably reflect the degree of arterial stiffness [5], is low-cost, easy to implement, and has cardiovascular risk predictive value [6, 7].

Insulin resistance (IR) is defined as a decrease in insulin responsiveness in tissues, and is an important factor in the glycolipid metabolic pathway [8]. Due to the production of vascular inflammatory factors and impairment of vascular endothelial smooth muscle cell function, IR is considered a major risk factor for the development of arterial stiffness and CVDs [9]. Currently, the hyperinsulinemic-euglycemic clamp (HEC) is the gold standard for assessing IR [10]. However, this traditional evaluation method is time-consuming, expensive, complicated, and labour-intensive, making it unsuitable for clinical use. Recently, various studies have shown that the TyG index, calculated from fasting triglyceride (TG) and fasting blood glucose (FBG) levels, can be used as a reliable and inexpensive surrogate biomarker of IR, with better performance than the homeostatic modelling assessment of IR (HOMA-IR) [11–13].

Studies have indicated that elevated TyG index levels are associated with an increased risk of arterial stiffness [14–16]. However, most studies were conducted in populations with underlying conditions (e.g. hypertension, insulin resistance, and diabetes mellitus) and involved small sample sizes. Therefore, there is a need for studies with large sample sizes to further validate this association. Limited knowledge exists regarding the role of the TyG index in assessing the risk of arterial stiffness in individuals with normoglycaemia, and previous studies have not used ePWV to measure arterial stiffness. Therefore, the present study used relevant databases to conduct an association analysis, aiming to elucidate whether the TyG index can be used as an independent biomarker for predicting the risk of arterial stiffness in individuals with normoglycaemia. The objective is to identify individuals at risk of arterial stiffness at an early stage, aiding clinicians in formulating more targeted preventive strategies and clinical interventions.

## Methods

### Study design and population

**Fig. 1:**
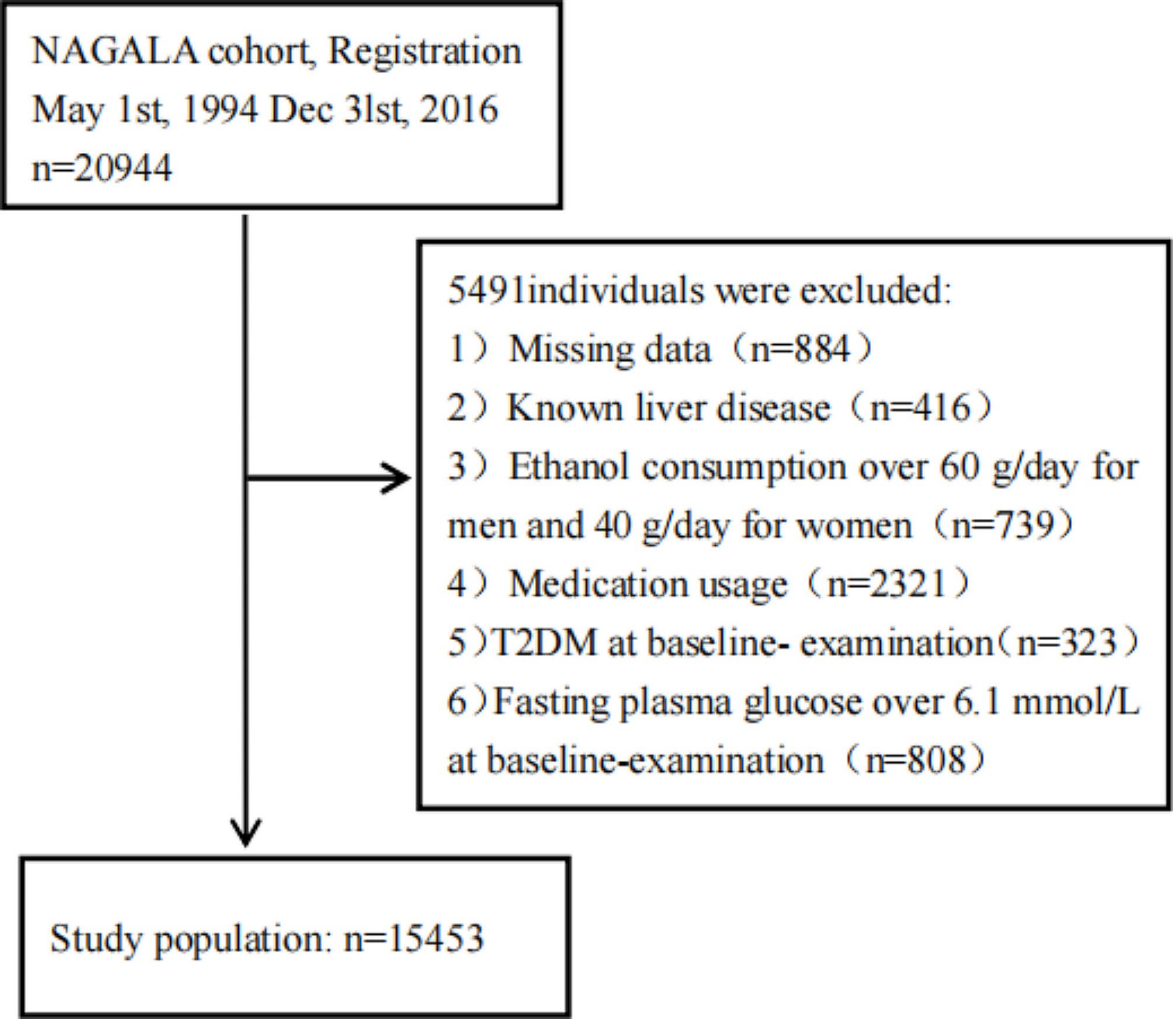
Flowchart of participants’ selection

This study used data from the DATADRYAD, of which a detailed description has been previously provided [17]. All researcher data are freely available from the website: http://www.Datadryad.org/. We used a standardised questionnaire to collect the following variables: sociodemographic information, health behaviours, medical history, and substance use. Subsequently, we measured physical parameters using standardised equipment, and collected serum specimens from the study participants after they fasted for 8 h. The biochemical parameters included ALT, AST, GGT, FPG, HDL-C, TC, TG, and HbA1c. A total of 20,944 participants were recruited between 2004 and 2015. The inclusion criteria comprised individuals with normal blood glucose levels (no history of diabetes or with a fasting blood glucose < 6.1 mmol/L at baseline examination) and with a complete blood pressure measurement data. The exclusion criteria were as follows: (1) missing recorded data, (2) known history of liver disease, (3) alcohol abuse (> 60 g/day for men and > 40 g/day for women), (4) history of oral medication use, and (5) history of diabetes or fasting blood glucose > 6.1 mmol/L at baseline. In total, 15,453 participants (8,419 women and 7,034 men) were included in the analysis (Fig. 1). The project was approved by the Ethics Committee of the Murakami Memorial Hospital and all participants signed a written informed consent form.

### Triglyceride-glucose (TyG) index

The TyG index was calculated as follows: Ln [fasting triglycerides (mg/dL) × fasting blood glucose (mg/dL)/2] [18].

### Estimating pulse wave velocity (ePWV)

It was calculated as follows: ePWV = 9.587 − 0.402 × age + 4.560 × 10 − 3 × age 2 − 2.621 × 10 − 5 × age 2 × MBP + 3.176 × 10 − 3 × age × MBP − 1.832 × 10 − 2 × MBP, and MBP was calculated as DBP + 0.4 (SBP−DBP) [19]. An ePWV ≥ 10 m/s was defined as arterial stiffness [20].

### Covariate assessment

We considered both sociodemographic characteristics and health-related factors. Sociodemographic characteristics included age and sex. Health-related factors included: history of alcohol consumption, as measured by alcohol breakdown and average weekly alcohol consumption in the previous month (none or minimal drinking (40 g/week), light drinking (40–140 g/week), moderate drinking (140–280 g/week), and heavy drinking (> 280 g/week)) [21]; smoking history (no smoking, smoking cessation, or current smoking); regular exercise, defined as regular participation in any type of sport more than once per week [22]; liver disease, meeting the diagnostic criteria in abdominal ultrasound [23]; obesity, defined as a body mass index (BMI) of ≥ 25 kg/m2 [24]; and visceral fat obesity, defined as a waist circumference ≥ 90 cm in men or ≥ 80 cm in women [25].

### Statistical analysis

We processed and analysed the data using SPSS 25.0 software and expressed continuous variables with normal distribution by mean ± standard deviation (SD). Comparisons between groups was made using t-test and continuous variables with skewed distribution by median ± interquartile range (IQR) and Wilcoxon rank sum test. Categorical variables were expressed as numbers (percentages, %), and compared between groups using the chi-square test. To study the significance of intergroup differences stratified by TyG index quartiles, the Kruskal–Wallis test or ANOVA was used. Finally, the correlation between the TyG index and arterial stiffness was analysed using logistic regression.

To assess the influence of potential effect confounders, we conducted subgroup analyses according to: age (< 65 versus ≥ 65 years); sex (female versus male); BMI (< 25 versus ≥ 25 kg/cm 2), WC (< 90 versus ≥ 90 cm for men and < 80 versus ≥ 80 cm for women); alcohol consumption history (no drinking or minimal drinking (40 g/week) versus light drinking (40–140 g/week) versus moderate drinking (140–280 g/week) versus heavy drinking (> 280 g/week); and smoking history (no smoking versus abstaining versus current smoking).

In all analyses, statistical significance was achieved at *p* < 0.05.

## Results

### Baseline characteristics of participants

As observed in the flowchart (Figs. 1), 15,453 participants were included in this study, of whom 7,034 (45.5%) were men and 8,419 (54.5%) were women. The mean ± SD age of all participants was 43.7 ± 8.9 years and the mean ± SD TyG index was 8.0 ± 0.6.

The clinical and biological characteristics of participants in the TyG index quartiles are listed in Table 1. Participants with a higher TyG index were older and exhibited elevated BMI and WC values. Additionally, a greater proportion of individuals in this category were currently smoking or consuming alcohol compared to those in the lowest quartile group. Significant differences in biological parameters were observed between groups. Participants in the highest TyG index quartile had significantly higher ALT, AST, GGT, TC, TG, FPG, SBP, and DBP levels than those in the lowest quartile. The TyG index was significantly lower in women and regular exercisers.

**Table 1:** Clinical characteristics of the study population according to TyG

Variables	Total (*n* = 15,453)	Q1 (*n* = 3855)	Q2 (*n* = 3870)	Q3 (*n* = 3862)	Q4 (*n* = 3866)	*p* value
Age, (years)	43. 7 ± 8.9	40.5 ± 8.2	43. 5 ± 8.9	45.0 ± 8.9	45. 8 ± 8. 7	< 0.001
Men, n (%)	7034 (45.5)	2894 (75.1)	2054 (53.1)	1402 (36.3)	684 (17.7)	< 0.001
Alcohol consumption, n (%)						< 0. 001
None	11,802 (76.4)	3347 (86.8)	3003 (77.6)	2850 (73.8)	2602 (67. 3)	
Light	1754 (11.4)	283 (7.3)	459 (11.9)	500 (12.9)	512 (13.2)	
Moderate	1357 (8.8)	187 (4.9)	299 (7.7)	367 (9.5)	504 (13)	
Heavy	540 (3.5)	38 (1)	109 (2.8)	145 (3.8)	248 (6.4)	
Smoking. status, n (%)						< 0. 001
Never	9027 (58.4)	2984 (77.4)	2452 (63.4)	2046 (53)	1545 (40)	
Past	2949 (19.1)	466 (12.1)	686 (17.7)	813 (21.1)	984 (25.5)	
Current	3477 (22.5)	405 (10.5)	732 (18.9)	1003 (26)	1337 (34.6)	
Habit of exercise, n (%)						0.002
No	12,747 (82.5)	3160 (82)	3131 (80.9)	3204 (83)	3252 (84.1)	
Yes	2706 (17.5)	695 (18)	739 (19.1)	658 (17)	614 (15.9)	
BMI, (kg/ m2)	22.1 ± 3.1	20.4 ± 2.4	21.4 ± 2.7	22.5 ± 3.0	24.1 ± 3.1	< 0.001
WC, (cm)	76.5 ± 9.1	70. 8 ± 7.0	74.2 ± 8.0	77.9 ± 8.5	82.9 ± 8.1	< 0.001
ALT, (IU/ L)	17.0 (13.0, 23.0)	14.0 (11.0, 17.0)	15.0 (12.0, 20.0)	18.0 (14.0, 24.0)	23.0 (17.0, 32.0)	< 0.001
AST, (IU/ L)	17.0 (14.0, 21.0)	16.0 (13.0, 19.0)	17.0 (14.0, 20.0)	17.0 (14.0, 21.0)	19.0 (16.0, 24.0)	< 0.001
GGT, (IU/ L)	15.0 (11.0, 22.0)	12.0 (10.0, 15.0)	14.0 (11.0, 18.0)	16.0 (12.0, 23.0)	22.0 (16.0, 35.0)	< 0.001
TC, (mg/ dL)	1982 ± 33 4	1817 ± 29 6	1936 ± 30 4	2024 ± 30 8	2151 ± 33 4	< 0.001
HDL-c, (mg/dL)	56.5 ± 15.6	65.7 ± 14.7	60.7 ± 14.8	54.2 ± 13.3	45.6 ± 11.4	< 0.001
TG, (mg/ dL)	80.8 ± 58.1	32.6 ± 8.4	54.2 ± 7.3	80.2 ± 11.3	156.1 ± 67.4	< 0.001
FBG, (mg/ dl)	93.0 ± 7.4	88.4 ± 6.7	91.9 ± 6.9	94.3 ± 6.6	97.2 ± 6.6	< 0.001
HbA1, (%)	5.2 ± 0.3	5.1 ± 0.3	5.2 ± 0.3	5.2 ± 0.3	5.2 ± 0.3	< 0.001
SBP, (mmHg)	114.5 ± 15.0	107. 7 ± 12.8	112.3 ± 14.1	116. 3 ± 14.4	121.7 ± 14.9	< 0.001
DBP, (mmHg)	71.6 ± 10.5	66.6 ± 9.1	69.9 ± 9.9	72.9 ± 10.0	76.9 ± 10.3	< 0.001
TyG	8.0 ± 0.6	7.2 ± 0.3	7.8 ± 0.1	8.2 ± 0.1	8.9 ± 0.3	< 0.001

TG, triglyceride; DBP, diastolic blood pressure; SBP: Systolic blood pressure; BMI: Body mass index; WC: Waist circumference; ALT: Alanine aminotransferase; ASL: Aspartate aminotransferase; GGT: Gamma glutamyl; TC: Total cholesterol; HDL-C, high-density lipoprotein cholesterol; HbA1c, haemoglobin A1c

### Univariate and multivariate analyses of arterial stiffness

**Fig. 2:**
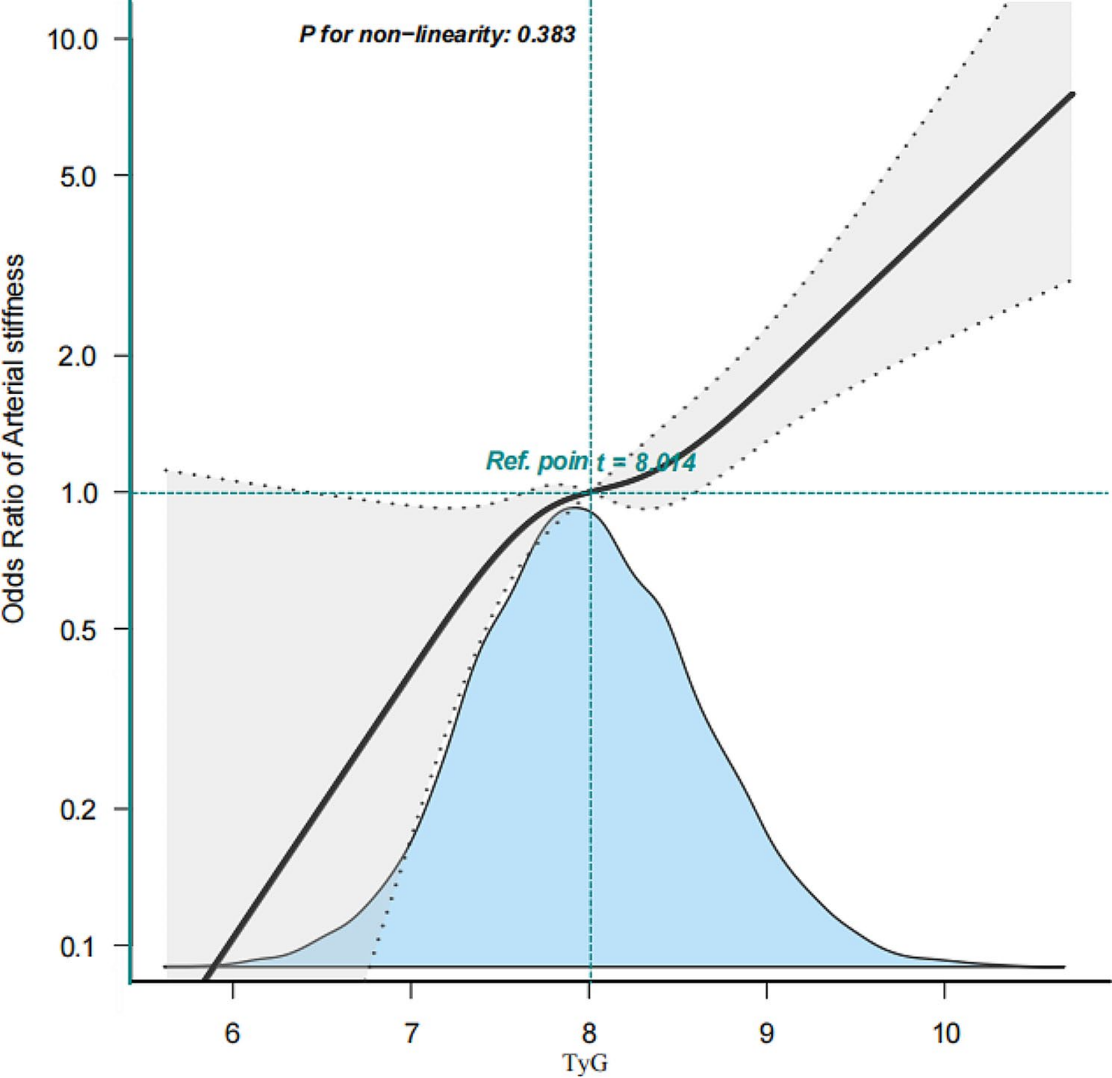
Associations between TyG index with arterial stiffness

Age, sex, alcohol consumption, smoking history, regular exercise, BMI, WC, ALT, AST, GGT, TC, HDL-c, TG, FPG, and HbA1c levels were significantly associated with arterial stiffness (Table 2). After adjusting for the covariates included in Table 2, the risk of developing arterial stiffness steadily increased when the TyG index was used as a continuous variable (Fig. 2).

**Table 2:** Results of univariate analysis of ePWV

Variable	OR 95% CI	*p* value
Age, (years)	1.32 (1.29–1.34)	< 0.001
Men, n (%)	2.49 (2.04–3.06)	< 0.001
Alcohol consumption, n (%)		
None	ref	
Light	1.71 (1.32–2.21)	< 0.001
Moderate	2.42 (1.89–3.11)	< 0.001
Heavy	2.77 (1.95–3.95)	< 0.001
Smoking. status, n (%)		
Never	ref	
Past	2.65 (2.17–3.23)	< 0.001
Current	1.04 (0.82–1.34)	0.732
Habit of exercise, n (%)	1.73 (1.41–2.11)	< 0.001
BMI, (kg/ m2) WC, (cm)	1.13 (1.11–1.16) 1.07 (1.06–1.08)	< 0.001 < 0.001
ALT, (IU/L)	1 (1–1.01)	0.045
AST, (IU/L)	1.02 (1.01–1.03)	< 0.001
GGT, (IU/L)	1.01 (1.01–1.01)	< 0.001
TC, (mg/dL)	1.01 (1.01–1.01)	< 0.001
HDL-c, (mg/dL)	0.99 (0.98–0.99)	< 0.001
TG, (mg/dL)	1.01 (1–1.01)	< 0.001
FBG, (mg/dl)	1.1 (1.08–1.11)	< 0.001
HbA1, (%)	4.49 (3.41–5.92)	< 0.001

TG, Triglyceride; DBP: Diastolic blood pressure; SBP: Systolic blood pressure; BMI: Body mass index; WC: Waist circumference; ALT: Alanine aminotransferase; ASL: Aspartate aminotransferase; GGT: Gamma glutamyl; TC: Total cholesterol; HDL-C: High-density lipoprotein cholesterol; HbA1c: Hemoglobin A1c

We used various linear regression models to analyse the association between the TyG index and arterial stiffness. After adjusting for the two variables of age and sex (model 1), the OR of the linear regression between the two was 1.92 (95% Confidence Interval (CI): 1.59–2.33; *p* < 0.001), and after continuing to add multiple variables of smoking history, alcohol consumption, regular exercise, waist circumference, BMI, ALT, TC, HDL-C, and HbA1 to the models (models 2–3), the corresponding ORs were 1.46 (95% CI: 1.18–1.79; *p* < 0.001) and 1.86 (95% CI: 1.45–2.39; *p* < 0.001). This suggests that the TyG index is independently and positively associated with arterial stiffness in all three models after adjusting for different covariates (Table 3).

**Table 3:** Multivariable-adjust ORs and 95% CI of the TyG quartiles associated with ePWV

Variable	Unadjusted		Model 1		Model 2		Model 3	
	OR (95% CI)	*p* value	OR (95% CI)	*p* value	OR (95% CI)	*p* value	OR (95% CI)	*p* value
TyG	2.33 (2.04–2.67)	< 0.001	1.92 (1.59–2.33)	< 0.001	1.46 (1.18–1.79)	< 0.001	1.86 (1.45–2.39)	< 0.001
1st Quartile (< 7.59)	1(Ref)		1(Ref)		1(Ref)		1(Ref)	
2st Quartile (7.59–8.01)	3.02 (2.06–4.41)	< 0.001	1.7 (1.08–2.68)	0.022	1.48 (0.93–2.37)	0.099	1.61 (1–2.59)	0.049
3st Quartile (8.01–8.45)	3.99 (2.76–5.77)	< 0.001	1.9 (1.22–2.94)	0.004	1.47 (0.94–2.32)	0.095	1.72 (1.07–2.75)	0.025
4st Quartile (≥ 8.45)	6.31 (4.42–9)	< 0.001	2.93 (1.91–4.5)	< 0.001	1.81 (1.16–2.84)	0.01	2.37 (1.44–3.9)	0.001
p for trend	1.66 (1.52–1.82)	< 0.001	1.38 (1.23–1.54)	< 0.001	1.16 (1.03–1.31)	0.013	1.28 (1.11–1.47)	0.001

Model 1 adjusted for age and sex

Model 2 adjusted for Model 1 + smoking status, alcohol consumption, habit of exercise, WC, and BMI.

Model 3 adjusted for Model 1 + Model 2 + ALT, TC, HDL-C, and HbA1.

The TyG index was transformed into categorical variables using quartiles as cutoffs, as follows: Q1 (< 7.95), Q2 (7.59–8.01), Q3 (8.01–8.45), and Q4 (≥ 8.45) for use in regression analyses with arterial stiffness. The results revealed that in the unadjusted model and the model adjusted for all variables of age, sex, smoking, alcohol consumption, regular exercise, waist circumference, BMI, ALT, TC, HDL-c, and HbA1 (model 3), the OR for Q2 was 3.02 (95% CI: 2.06–4.41; *p* < 0.001), 1.61 (95% CI: 1–2.59; *p* = 0.049), and 3.99 (95% CI: 2.76–5.77; *p* < 0.001) for Q3, compared to Q1, respectively. Additionally, the OR was 1.72 (95% CI: 1.07–2.75; *p* = 0.025) for Q4 in the fully adjusted model, and ORs of 6.31 (95% CI: 4.42–9; *p* < 0.001) and 2.37 (95% CI: 1.44–3.9; *p* = 0.001) in the unadjusted and fully unadjusted models, respectively. The trend test was significant in both models (*p* < 0.001), indicating a stable linear association between TyG index and arterial stiffness (Table 3).

### Subgroup analyses by adjusted potential effect confounders

**Fig. 3:**
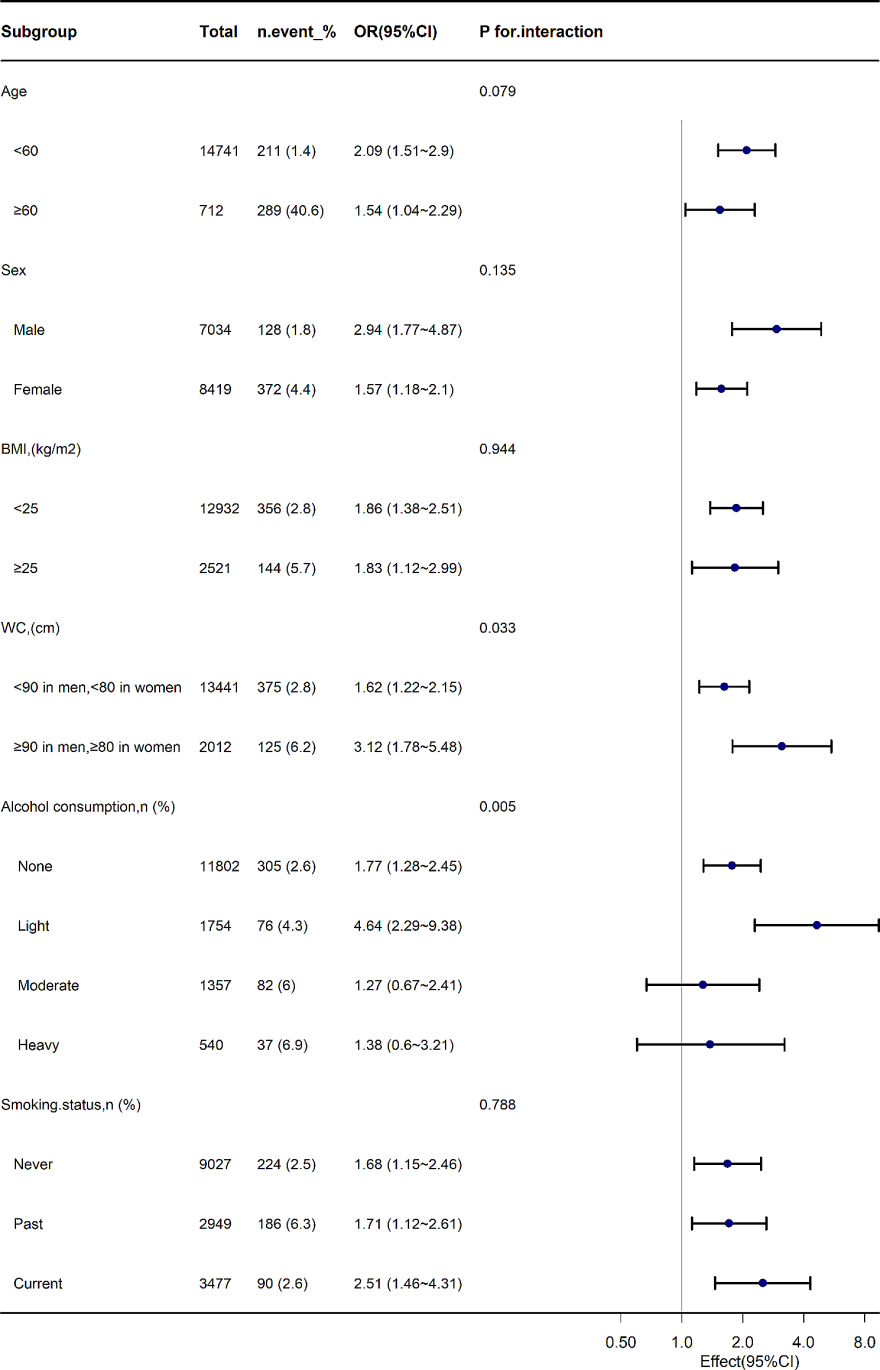
Subgroup analysis of the TyG and ePWV

Subgroup analyses were performed to assess the effect of the TyG index (per 1-unit increment) on arterial stiffness in the different subgroups (Fig. 3). As observed in the forest plot, the positive association between the TyG index and arterial stiffness was stronger in the WC and alcohol consumption groups (P interaction = 0.033 and 0.005, respectively). In contrast, the association between the TyG index and arterial stiffness was not significantly altered in the age, sex, BMI, and smoking groups.

## Discussion

In this large cross-sectional survey based on a Japanese population with normoglycaemia, after rigorous adjustment for covariates, we found that the TyG index was positively associated with arterial stiffness, both as a continuous and categorical variable with quartile cutoff points. The higher the TyG index, the higher the risk of arterial stiffness.

To the best of our knowledge, few studies have examined the association between TyG index and arterial stiffness in populations with normoglycaemia, and even fewer have evaluated arterial stiffness using ePWV. However, assessment of arterial stiffness using ePWV suggests that an association between the TyG index and arterial stiffness has been reported in several studies [11, 26–28]. Lambrinoudaki et al. [29] found an association between the TyG index and arterial stiffness, assessed using PWV between the common carotid and common femoral arteries. However, this study has limitations due to its small sample size and inclusion of only women during post-menopause and without diabetes. Another study by Lee et al. [11] found an association between the TyG index and an increased risk of arterial stiffness, independently assessed using PWV in healthy Korean adults. Nonetheless, this study did not exclude individuals with diabetes and an unquantifiable history of alcohol consumption. Similarly, Wu et al. [14] discovered a positive association between the TyG index and the risk of arterial stiffness progression, as assessed by brachial-ankle PWV. Despite indicating that a higher TyG index predicted an increased risk and faster progression of arterial stiffness, this study, like Lee et al.‘s [11], did not exclude individuals with diabetes and had a small sample size. Since diabetes mellitus is a major risk factor of arterial stiffness, the results of these studies may be influenced by the presence of diabetes mellitus. In our study, we report, for the first time, the correlation between the TyG index and arterial stiffness in a population with normal blood glucose levels. Our findings clearly demonstrate that the TyG index is independently and positively correlated with ePWV, further confirming its potential as a novel and simple non-invasive biomarker for predicting the risk of arterial stiffness.

Some researchers have suggested that risk factors such as age, sex, BMI, WC, alcohol consumption, and smoking history may confound the association between the TyG index and atherosclerosis [11–15]. However, we performed subgroup analyses adjusting for potential effect confounders, and the results indicated that only the inclusion of WC and alcohol consumption significantly altered the association between TyG index and arterial stiffness. In contrast, factors such as age, gender, BMI, and smoking history showed no significant impact. The conflicting results may be attributed to differences in age and body composition, which require further validation.

The underlying biological mechanisms of the association between TyG index and arterial stiffness remain unclear. We postulate that these mechanisms may be related to metabolic pathways, inflammation, endothelial dysfunction, or other factors influenced by TyG dysregulation. Prolonged impairment of glucose metabolism has been associated with increased aortic stiffness [30], and IR and IR-related metabolic disorders have been linked to the development of arterial stiffness. Studies have demonstrated that IR contributes to the development of arterial stiffness through the following pathways: (1) IR can disrupt insulin signalling at the level of endothelial cells, vascular smooth muscle cells, and macrophages leading to varying degrees of oxidative stress and impaired endothelial cell function; this, in turn reduces nitric oxide bioavailability [31], causes vascular functional and structural damage, and ultimately reduces arterial wall dilatancy, leading to arterial stiffness [32]; (2) IR promotes the development of atherosclerotic dyslipidaemia, increases the vascular inflammatory response, disrupts endothelial function, and influences the prethrombotic state and arterial stiffness [33]; and (3) IR accelerates the accumulation of advanced glycosylation end products (AGEs), alters collagen and elastin contents, and remodels the arrangement and structure of the extracellular matrix, which in turn induces changes in arterial stiffness [33, 34].

In addition, systemic inflammation plays a role in atherosclerotic process [35]. C-reactive proteins have direct pro-inflammatory effects on human endothelial cells [36] and can induce endothelial dysfunction [37], and endothelial-derived nitric oxide is important for the functional regulation of arterial stiffness in large arteries in vivo [38, 39]. Alternatively, inflammation may induce structural changes in the arterial wall by altering the balance between elastin breakdown and synthesis [35]. In conclusion, the specific mechanisms of TyG index and arterial stiffness needs to be investigated further.

Our study has the following advantages: first, the data analysis was based on a large sample size, and the results of the study are stable and reliable; second, our study population included a wide range of adults with normal glycaemia, which is more representative of the population; and lastly, our assessment method is simple, low-cost, and can be widely used in clinical practice. However, our study has some limitations: first, this study has cross-sectional design, which does not allow us to verify the causal relationship; second, the study data were from the Japanese population, which has ethnic group specificity; and third, we were limited by the database and could not compare the TyG index with the HOMA-IR and HEC trials. Despite these limitations, our study provides reliable evidence of the association between the TyG index and arterial stiffness, further deepens the understanding of the TyG index in predicting arterial stiffness in individuals with normoglycaemia and provides a direction to guide the development of more targeted preventive strategies and clinical interventions.

## Conclusion

The TyG index significantly correlated with arterial stiffness, suggesting that it can be used as a predictor of early arterial stiffness. The TyG index should generally be applied to screen for arterial stiffness in clinical practice and to perform individualised prevention and management at an early stage, which will help reduce the incidence of CVDs.

## Data Availability

The datasets used and/or analysed in the current study are available from the corresponding author upon reasonable request.

## Abbreviations

TyG: Triglyceride-glucose
CVDs: Cardiovascular diseases
IR: Insulin resistance
SMC: Smooth muscle cell
HEC: High insulin normoglycaemic clamp
HOMA-IR: Homeostasis model assessment of insulin resistance
FBG: Fasting blood glucose
TG: Triglyceride
PWV: Pulse wave velocity
AASI: Ambulatory arterial stiffness index
ePWV: Estimated pulse wave velocity
MBP: Mean blood pressure
DBP: Diastolic blood pressure
SBP: Systolic blood pressure
BMI: Body mass index
WC: Waist circumference
ALT: Alanine aminotransferase
ASL: Aspartate aminotransferase
GGT: Gamma glutamyl
TC: Total cholesterol
HDL-C: High-density lipoprotein cholesterol
HbA1c: Hemoglobin A1c
SD: Standard deviation
IQR: Interquartile range
AGEs: Advanced glycosylation end products
